# Does ecophysiology mediate reptile responses to fire regimes? Evidence from Iberian lizards

**DOI:** 10.7717/peerj.2107

**Published:** 2016-06-09

**Authors:** Catarina C. Ferreira, Xavier Santos, Miguel A. Carretero

**Affiliations:** 1 Departamento de Biologia, Faculdade de Ciências da Universidade do Porto, Porto, Portugal; 2 CIBIO Research Centre in Biodiversity and Genetic Resources, InBIO, Universidade do Porto, Vairão, Portugal

**Keywords:** Reptiles, Habitat, Functional response, Preferred temperatures, Water-loss rates, Wildfires

## Abstract

**Background.** Reptiles are sensitive to habitat disturbance induced by wildfires but species frequently show opposing responses. Functional causes of such variability have been scarcely explored. In the northernmost limit of the Mediterranean bioregion, lizard species of Mediterranean affinity (*Psammodromus algirus* and *Podarcis guadarramae*) increase in abundance in burnt areas whereas Atlantic species (*Lacerta schreiberi* and *Podarcis bocagei*) decrease. *Timon lepidus*, the largest Mediterranean lizard in the region, shows mixed responses depending on the locality and fire history. We tested whether such interspecific differences are of a functional nature, namely, if ecophysiological traits may determine lizard response to fire. Based on the variation in habitat structure between burnt and unburnt sites, we hypothesise that Mediterranean species, which increase density in open habitats promoted by frequent fire regimes, should be more thermophile and suffer lower water losses than Atlantic species.

**Methods.** We submitted 6–10 adult males of the five species to standard experiments for assessing preferred body temperatures (*T*_*p*_) and evaporative**water loss rates (EWL), and examined the variation among species and along time by means of repeated-measures AN(C)OVAs.

**Results.** Results only partially supported our initial expectations, since the medium-sized *P. algirus* clearly attained higher *Tp* and lower EWL. The two small wall lizards (*P. bocagei* and *P. guadarramae*) displayed low *Tp* and high EWL while the two large green lizards (*T. lepidus* and *L. schreiberi*) displayed intermediate values for both parameters.

**Discussion.** The predicted differences according to the biogeographic affinities within each pair were not fully confirmed. We conclude that ecophysiology may help to understand functional reptile responses to fire but other biological traits are also to be considered.

## Introduction

Wildfire is considered among the environmental disturbances with a major impact on ecosystem functioning and composition in many areas of the world (Bond, Woodward & Midgley, 2005). Global climate change (Piñol, Terradas & Lloret, 1998; McKenzie et al., 2004; Kasischke & Turetsky, 2006; Westerling et al., 2006) and shifts in land-use practices (e.g., agricultural abandonment and urban sprawl; Moreira, Rego & Ferreira, 2001; Moreira & Russo, 2007) are considered primary causes for the recent increase of fire frequency and extension. Changes in fire regimes are expected to provoke profound effects on the biodiversity and composition of local communities (McKenzie et al., 2004). The increase of fire risk and activity in recent decades (Pausas & Fernández-Mun¯oz, 2012) has attracted considerable interest mainly addressed to understanding the environmental drivers and effects of fire, especially in the context of global change (Bowman et al., 2009; Flannigan et al., 2009; Whitlock et al., 2010). In the context of a present shift in fire regimes, a pressing question is whether we are prepared to manage fire regimes and reduce impacts of fire on many ecosystem components (Pausas & Keeley, 2009). An ecologically-based framework is crucial in the 21st century conservation scenario (Nimmo et al., 2015) in order to understand how species respond to fire and what is the resistance and resilience of communities towards this disturbance.

The response of species to fire is largely driven by habitat structure (Santos, Badiane & Matos, 2016) with some species selecting early or late post-fire stages following a continuum along vegetation succession (Letnic et al., 2004; Santos & Poquet, 2010; Valentine et al., 2012; Santos, Badiane & Matos, 2016). The ‘habitat accommodation’ model of succession proposed by Fox (1982), applied to fire ecology, represents a useful framework to help understand and predict the response of animals to fire. However, field-based studies have failed to support this model (Driscoll & Henderson, 2008; Lindenmayer et al., 2008) since the responses of species to fire vary in space and time (Driscoll et al., 2012; Nimmo et al., 2012; Smith, Bull & Driscoll, 2013) due to the complexity of biotic and abiotic interactions between vegetation dynamics, animal species, and climate (Nimmo et al., 2014). For this reason, to improve predictive models of fire responses in animal communities, detailed ecological (functional) information on species is needed (Smith, Bull & Driscoll, 2013).

Functional approaches have gained acceptance in community ecology due to the possibility of quantification and predictive power (McGill et al., 2006). Although such analyses have rarely been applied to fire ecology (i.e., traits favoured in burnt areas), some recent studies highlight its importance for predicting the responses of reptiles to fire (see references above). Reptiles are suitable organisms to examine responses to fire following parallel habitat-based and functional approaches. This is due to their sedentary behaviour, dependence on environment temperature and strait association with habitat (Huey, 1982). While reptile responses to fire are often considered to be habitat-mediated, such association is not necessarily direct. For instance, since fire opens the habitat in the short term, many reptiles are expected to benefit from the thermal quality of open areas created by fire (Bury, 2004). Different species, however, display opposing responses depending on their habitat preferences, biogeographic affinities and life-history traits (Pastro, Dickman & Letnic, 2013; Smith, Bull & Driscoll, 2013; Ferreira, Mateus & Santos, 2016; Santos, Badiane & Matos, 2016). For example, in a mixed reptile community composed of Mediterranean and non-Mediterranean elements, Santos & Cheylan (2013) detected that repeated-fire regime favoured reptile assemblages composed of Mediterranean species with short lifespan and insectivorous habits.

As ectothermic and sedentary animals, terrestrial reptiles are directly and locally exposed to environmental variation in temperature and humidity which makes them ideal subjects for mechanistic ecological modelling (Kearney & Porter, 2009). Specifically, their intrinsic physiological features may potentially govern the response of different species to fire. Thus, body temperature is considered the most important environmental variable affecting performance of ectotherms, including reptiles (Angilletta, 2010), heat balance resulting from thermal characteristics of the environment (Porter et al., 1973; Porter & Tracy, 1983). In particular, thermoregulatory reptiles respond to thermal heterogeneity by selecting microhabitats with adequate temperatures and modifying their body postures to maximize heat gain or loss. However, during these processes reptiles also suffer evaporative water loss (EWL) mainly through the skin but also through respiratory passages and the eyes (Shoemaker & Nagy, 1977; Mautz, 1982a). Since evaporation increases with temperature, a trade-off between thermoregulation and water balance in ectotherms has been suggested (Mautz, 1982a). EWL could hence represent a constraint for the activity of ectotherms when water is not available. In this context, reptiles exposed to unburnt and burnt habitats are expected to face opposing environmental conditions. Under similar climate regimes, unburnt habitats provide more abundant and complex vegetation compared to that in open burnt habitats that in turn are more exposed to extreme temporal variations in temperature and humidity (Ferreira, 2015; data from NW Portugal available from the authors upon request). Certainly, environmental constraints may preclude reptiles from attaining their physiological optima (i.e., Gvoždík, 2002) but under the same conditions some species will be further from their optima than others (Grbac & Bauwens, 2001). Even if such species may still survive under suboptimal conditions in a complex landscape, their fitness will be affected and demographic repercussions are expected (Castilla, Van Damme & Bauwens, 1999). Thus, we hypothesize that reptile species prevailing in unburnt (forested) habitats will maintain lower and less variable body temperatures and experience lower water loss rates as vegetation buffers daily variation of humidity and temperature (Ferreira, 2015). In contrast, reptiles inhabiting burnt (open) habitats will attain higher but temporally variable body temperatures and suffer higher evaporative water stress.

To test this hypothesis we analysed the community of lacertid lizards inhabiting Northern Portugal, a transition zone between Atlantic and Mediterranean bioregions, where species of different biogeographic affinities coexist. Lacertids constitute a guild of diurnal, heliothermic and mainly insectivorous lizards, highly diversified in habitat use (Arnold, 1987), which dominate reptile assemblages across the Mediterranean basin (Carretero, 2004). In syntopy, species from both biogeographic affinities spatially segregate in a gradient from forested to open microhabitats within patchy and heterogeneous landscapes (Ferreira, Žagar & Santos, in press). Results from systematic monitoring during the last decade in burnt and unburnt areas in Northern Portugal and other areas at the northernmost limit of the Mediterranean bioregion agree with microhabitat segregation at a smaller scale. We detected opposing responses to fire by different species mostly related to their biogeographic affinity. Namely, Mediterranean species *Psammodromuns algirus* and *Podarcis guadarramae* increased in abundance in burnt areas whereas the Atlantic species *Lacerta schreiberi* and *Podarcis bocagei* decreased (Santos & Poquet, 2010; Santos & Cheylan, 2013; Ferreira, Mateus & Santos, 2016). The Mediterranean lizard *Timon lepidus* displayed mixed responses depending on the population. Whereas in the northernmost limit of its distribution, it was favoured by fire (Santos & Cheylan, 2013), in Mediterranean habitats it prefers long-term unburnt sites suggesting negative short-term responses to fire (Santos, Badiane & Matos, 2016). Based on this empirical evidence, we conducted a comparative experimental study in order to determine whether Mediterranean and Atlantic species responses to fire are correlated to their thermal and hydric physiology. Two ecophysiological traits were selected to represent the general species’ trends: preferred body temperature and evaporative water loss. Specifically, we tested whether species favoured by repeated fire regimes would be more thermophile and achieve lower water loss rates than those negatively affected. In this case, we predict that *T. lepidus*, *P. algirus* and *P. guadarramae*, species that respond positively, should have higher preferred temperatures and lower evaporative water loss than *L. schreiberi* and *P. bocagei*, species that respond negatively.

## Materials and Methods

### Study area

The Iberian Peninsula encompasses a transition region between the Euro-Siberian and Mediterranean biogeographic regions (Metzger et al., 2005; Soares & Brito, 2007). These biogeographic crossroads are known as areas of high diversity of species and habitats (Spector, 2002). The extensive contact between Mediterranean and Atlantic climates leads to a high biodiversity of plants and animals, due to the co-existence of Atlantic and Mediterranean typical species in sympatry (Araújo, Thuiller & Pearson, 2006; Sillero et al., 2009). One of these transitional areas is found in Northern Portugal (Soares et al., 2005). This is one of the areas in Europe with the highest amount of burnt land; wildfire is considered a fundamental agent of landscape change (Silva et al., 2011). Transitional zones affected by intense fire history provide an opportunity to compare the ecological trends of both types of species, either in a general conservation context (Kati et al., 2004) or more specifically to make predictions on the effects of wildfires on herpetofauna biodiversity (Hooper et al., 2005).

### Species sampling

The five lacertid species used in physiological experiments are the reptiles most frequently found in the area (Loureiro et al., 2008). These species vary in terms of body size, habitat preferences, distribution, biogeographic affinities and response to fire (Table 1).

**Table 1:** General traits of the five lizard species studied. Habitat, distribution and biogeographic affinities after Carretero, Galán & Salvador (2015), Galán (2015), Kaliontzopoulou et al., (2011) and Loureiro et al. (2008). Fire responses after (Santos & Cheylan, 2013; Santos & Poquet, 2010); and Ferreira, Mateus & Santos (2016).

Species	Body size	Main habitat	Distribution	Biogeography	Fire response
*Timon lepidus*	Very large	Open oak forest, big rocks	Iberia, SW France	Mediterranean	Increase
*Lacerta schreiberi*	Large	Ecotones and riverine forests	W Iberia	Atlantic	Decrease
*Psammodromus algirus*	Medium	Shrubs	Iberia, SW France, N Africa	Mediterranean	Increase
*Podarcis bocagei*	Small	Dunes, grasslands, walls, rocks	NW Iberia	Atlantic	Decrease
*Podarcis guadarramae*	Small	Rocks, crevices	W and central Iberia	Mediterranean	Increase

Lizards used in experiments were captured with a noose (Garcí a-Muñoz & Sillero, 2010) in three areas: *P. algirus* were captured in Serra da Estrela (40°51′N, 7°53′W), *T. lepidus* and *L. schreiberi* in Vairão (41°32′N, 8°67′W) and, finally, *P. bocagei* and *P. guadarramae* syntopic in Moledo (41°84′N, 8°87′W). All the sites are located in Northern Portugal and selected according to the availability of lizards.

To exclude effects of reproduction, body condition and ontogeny on *T*_*p*_ (Carretero, Roig & Llorente, 2005) we only captured 6–10 adult males during the peak of the reproductive season (May), which were kept in individual cages before the experiments, with water and food provided *ad libitum*. We measured body mass (BM) to the nearest 0.0001 g of each lizard with a digital balance and snout–vent lengths (SVL) to the nearest 0.01 mm with a digital calliper. After a short period of acclimation (1–2 days) to prevent lizards losing body condition (Brown & Griffin, 2003) we submitted them to temperatures and water loss experiments in two consecutive days and released them at their capture sites after the experiments had finished and after being fed and rehydrated.

The Institute for the Conservation of Nature and Forest (ICNF, Portugal) granted sampling permit (no. 459I2015ICAPT). Experiments followed the ethical guidelines of University of Porto.

### Preferred temperatures

Preferred body temperature (*T*_*p*_, body temperature achieved in the absence of thermoregulatory constraints; Huey & Bennett, 1987) provides a reliable representation of the overall thermal requirements of a given species while holding some logistic advantages. Namely, (1) it correlates directly with several physiological optima (Bauwens et al., 1995); (2) its temporal variation is relatively narrow in lacertids, which are known to be good thermoregulators (Huey, 1982); (3) it displays phylogenetic signal (Bauwens et al., 1995) and remains conservative in conspecific populations under different climate regimes in many lacertid species (i.e., Díaz, Iraeta & Monasterio, 2006); and (4) it can be reliably recorded in the lab under standardised conditions (Osojnik et al., 2013). It is true that at the individual level, *T*_*p*_ may change as a function of time of the day, season, feeding activity, ontogeny or reproductive status (Castilla, Van Damme & Bauwens, 1999) but these biases can be removed by restricting comparisons to a single class (i.e., adult males) and time period (i.e., spring) following a strict experimental protocol (Carretero, Roig & Llorente, 2005).

Lizards were individually exposed to a photothermal gradient between 20 and 50 °C produced by a 150-W infrared bulb fixed at one end of the terrarium (1 × 0.3 × 0.4 m) (Veríssimo & Carretero, 2009). The whole experiment was conducted in a room with temperature maintained at 22 °C to prevent wind and direct sun from affecting the temperatures in the terrarium while being exposed to natural photoperiod through a window. For every day of experimentation we turned on the bulb 1 h before the lizards were moved from the cages to the terrariums, and the first observation was made at 8 h.

The whole experiment was conducted between 8 h and 19 h, local summer time, the period of daily activity of the five species. At consecutive hourly intervals, we measured the body temperature of each lizard (precision ± 0.1 °C) using a k-thermocouple probe associated with a digital portable thermometer HIBOK 14 inserted into the animals cloaca (Veríssimo & Carretero, 2009). This standard procedure (Garcí a-Muñoz & Carretero, 2013; Osojnik et al., 2013) is a compromise between invasiveness and accuracy since at least the two *Podarcis* sp. and *P. algirus* are too small to allocate permanent cloacal probes or to implant transmitters (Clusella-Trullas et al., 2007), and too slender to provide accurate infrared readings (Carretero, 2012). While these latter methods could have been used with *T. lepidus* and probably with *L. schreiberi*, we preferred to keep the same measuring procedure for all five species for comparative purposes.

### Water-loss rates

Although reptilian hydric ecophysiology is less studied, evaporative water loss (EWL) rates are known to differ between species from xeric and humid habitats (Mautz, 1982b; Eynan & Dmi’el, 1993; Carneiro et al., 2015; Rato & Carretero, 2015) and between phylogenetically distant species in the same locality (Garcí a-Muñoz & Carretero, 2013; Osojnik et al., 2013). This makes EWL potentially informative on the hydric constraints of a species’ fundamental niche, particularly under restricted water availability (Bowker, 1993).

Water-loss experiments were always performed the day after the preferred temperature experiments when lizards were kept rehydrated in the terraria. We placed the lizards in closed cylindrical plastic boxes (9 cm diameter, 10 cm height) with ventilation holes in the top and at the bottom. Then, in groups of five boxes, lizards were placed into a bigger, opaque sealed chamber (40 × 30 × 20 cm) in dry conditions guaranteed by silica gel. Silica gel (∼100 g) was placed in a bag made of gauze and fixed with tape on the bottom of the chamber lid. In the same way, 5 g silica gel was placed at the bottom of each box containing a lizard. The amount of silica gel used guaranteed a low relative humidity inside of each box (20–25%). The experiment ran from 8 a.m. to 8 p.m. Conditions inside the chamber were monitored with a Fluke 971 temperature humidity meter (Fluke Corporation, Everett, Washington) at hourly intervals to ensure stability around ∼24 °C and 20–30% relative humidity. The environmental temperatures represented the lowest activity temperatures recorded for most lacertids in the field (Castilla, Van Damme & Bauwens, 1999) to prevent lizard stress inside the chambers while still providing relevant EWL rates. Every hour, the lizards were individually removed from the chamber, weighted using an analytical balance (precision ± 0.0001 g; CPA model 224S, Sartorius), and immediately placed back inside their respective boxes in the chamber.

### Statistical analyses

Once ensured that the distribution of *Tp* and EWL values did not deviate from normality (Shapiro–Wilk’s test, *P* > 0.05 in all cases) and the sphericity assumption was met (Mauchly’s tests *P* > 0.05), analyses for dependent measures were applied since both *Tp* and EWL were recorded for the same individual lizards through time. We first used analyses of (co)variance with repeated measures (AN(C)OVA-rm) to ascertain variation in *T*_*p*_ as a function of species and time interval (within- subject factor). In a second step, lizards’ SVL and body mass were incorporated as covariates to account for the effect of lizard size and shape (Carretero, Roig & Llorente, 2005). When significant, post hoc Duncan’s tests were performed between species pairs to detect eventual significant differences. For water loss experiments, we also used AN(C)OVA-rm to determine differences in instantaneous water loss (EWL_*i*_ = [(*W*_*n*_ − *W*_*n*+1_)∕*W*_0_] where *W* is the weight) between species and hour intervals, adding lizards’ SVL and body mass as covariates. We also calculated the accumulated water loss for the 11 intervals (EWL*a* = [(*W*_0_ − *W*_*n*_)∕*W*_0_] where *W* is the weight) and compared it between species using AN(C)OVA, also with SVL and body mass as covariates. The interaction between the mean *T*_*p*_ (calculated from 10 time interval measurements), BM, SVL and the total amount of water lost after the 12-hour experiment (EWL_*t*_ = [(*W*_0_ − *W*_11_)∕*W*_0_]) was assessed by standard multiple regression between species (average of *T*_*p*_ and EWL_*t*_ by species) and within species. All the analyses were performed in Statistica 12 (Statsoft, Tulsa, OK, USA; http://www.statsoft.com).

## Results

The five lizards species (Table 2) differed in SVL (ANOVA *F*_4,36_ = 176.55; *P* < 10^−6^) and body mass (ANOVA *F*_4,36_ = 151.26; *P* < 10^−6^). The post-hoc comparisons (Duncan tests *P* < 0.05) confirmed that the two *Podarcis* species were shorter and lighter, followed by *P. algirus*, then *L. schreiberi* and, finally, *Timon lepidus*, the longest and heaviest species. We also detected interspecific differences in robustness (ANCOVA on mass with SVL as covariable; *F*_4,35_ = 21.69; *P* < 10^−6^), *T. lepidus* being the most robust, followed by *P. bocagei*, *P. guadarramae* and *P. algirus*, and finally by *L. schreiberi,* which has the most elongated body.

**Table 2:** Number of adult males tested (*n*), snout-to-vent length (SVL), preferred body temperature (individual mean of 10 time intervals, *T*_*p*_), body mass (BM) and accumulated water loss (within 12 h, EWL_*t*_) for the five lizard species.

		SVL (mm)	*T*_*p*_(°C)	BM (g)	EWL_*t*_
Species	*n*	Mean ± SE Min–Max	Mean ± SE Min–Max	Mean ± SE Min–Max	Mean ± SE Min–Max
*T. lepidus*	6	140.72 ± 4.09	32.7 ± 0.3	69.7208 ± 5.2468	0.0097 ± 0.0012
		131.66–158.17	31.3–33.4	55.8682–85.7488	0.0057–0.0130
*L. schreiberi*	8	95.99 ± 4.43	33.4 ± 0.4	23.5756 ± 2.5086	0.0096 ± 0.0012
		71.66–111.45	32.3–35.3	10.0750–31.9960	0.0072–0.0162
*P. algirus*	8	74.38 ± 1.39	35.0 ± 0.1	11.5727 ± 0.9481	0.0038 ± 0.0006
		68.00–80.00	34.5–35.7	7.3401–15.1519	0.0020–0.0063
*P. bocagei*	10	54.04 ± 1.21	30.7 ± 0.3	3.5832 ± 0.1919	0.0299 ± 0.0024
		49.64–60.54	29.4–32.5	3.0369–4.8491	0.0235–0.0499
*P. guadarramae*	9	53.73 ± 0.93	31.5 ± 0.2	3.0744 ± 0.1391	0.0249 ± 0.0028
		49.88–58.57	30.7–32.2	2.3594–3.7809	0.0120–0.0377

### Preferred temperatures

**Figure 1:**
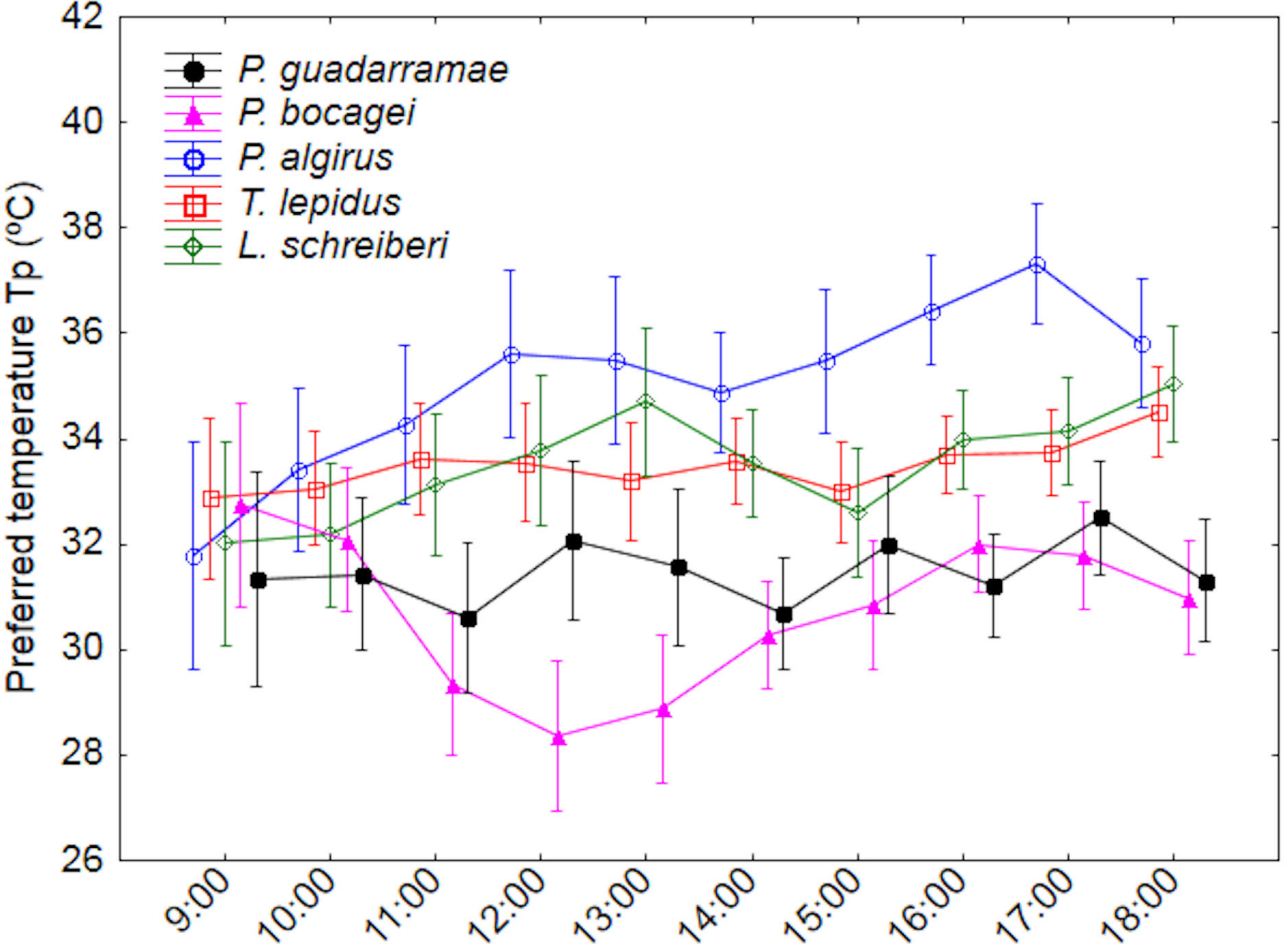
Daily variation of the preferred body temperatures (*T*_*p*_) for five lizard species. Median values and 0.95 confidence intervals are displayed.

We recorded variation in *T*_*p*_ between species, time intervals and time profile by species (interaction) (Tables 2 and 3; Table S1). Essentially, *P. algirus* selected higher *T*_*p*_ than all other species (Duncan tests *P* < 0.05), of which *L. schreiberi*, *T. lepidus*. *P. gaudarramae* and *P. bocagei* selected temperatures in gradually decreasing order. Such pattern remained when SVL and BM were used as covariates (Table 3). While time and time*species variation were also observed, patterns were complex (Fig. 1). Only *P. bocagei* displayed a clear bimodal variation with higher *T*_*p*_ selected in the early morning and late afternoon, the other species only showing irregular profiles. Finally, the significant interaction between time and the covariables indicated that *T*_*p*_ tended to show stronger temporal fluctuations in small lizards (Table 3).

**Table 3:** AN(C)OVA-rm of preferred temperatures (*T*_*p*_) and evaporative water loss rates (instantaneous, EWL_*i*_ and accumulated, EWL_*i*_) between the five lizard species for 10 and 11 consecutive hours, respectively; in the ANCOVA-rm we used snout-vent length (SVL) and body mass (BM) as covariables.

	*T*_*p*_			EWL_*i*_			EWL_*a*_		

	*d*.*f*.	*F*	*P*	*d*.*f*.	*F*	*P*	*d*.*f*.	*F*	*P*
ANOVA-rm									
Species	4, 36	37.42	<10^−6^	4, 36	13.27	10^−6^	4, 36	30.00	<10^−6^
Time	9, 324	5.42	10^−6^	10, 36	3.52	0.0002	10, 360	141.50	<10^−6^
Time*species	36, 324	2.73	10^−6^	40, 360	1.67	0.23	40, 360	19.07	<10^−6^
ANCOVA-rm (SVL, BM)									
SVL	1, 34	0.17	0.68	1, 34	0.23	0.64	1, 34	0.50	0.49
BM	1, 34	0.69	0.41	1, 34	0.03	0.86	1, 34	0.23	0.64
Species	4, 34	21.45	<10^−6^	4, 34	6.48	0.0005	4, 34	9.73	2*10^−5^
Time	9, 306	1.83	0.06	10, 340	0.57	0.84	10, 340	0.79	0.64
Time*SVL	9, 306	2.23	0.02	10, 340	0.57	0.83	10, 340	0.14	0.99
Time*BM	9, 306	1.96	0.04	10, 340	0.36	0.96	10, 340	0.09	0.99
Time*species	36, 306	1.53	0.03	40, 340	0.97	0.52	40, 340	6.97	<10^−6^

### Water-loss rates

**Figure 2:**
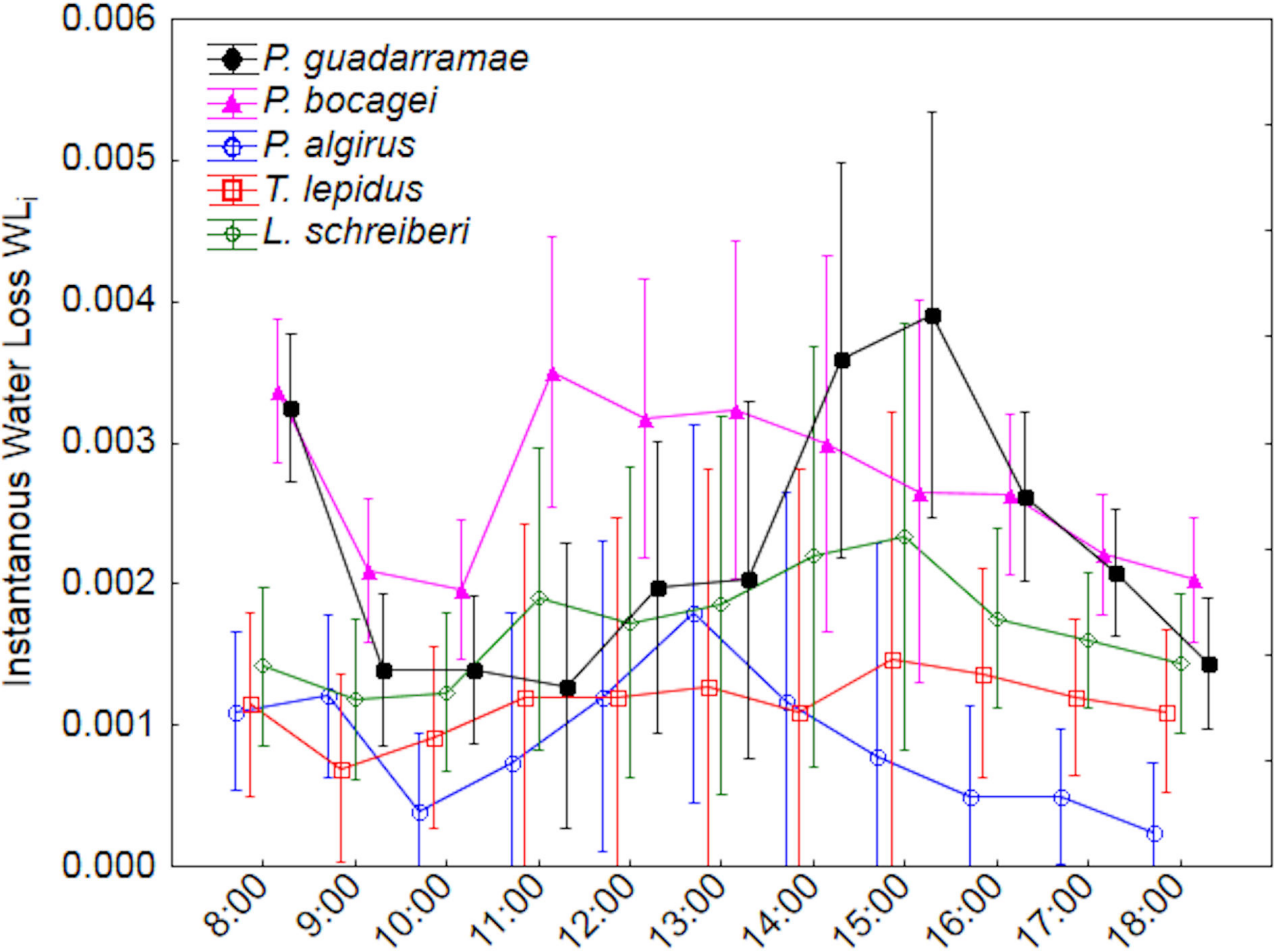
Patterns of instantaneous water loss (EWL_*i*_) along a 12-hour experiment for five lizard species. Median values and 0.95 confidence intervals are displayed.

Using ANOVA-rm, we also uncovered significant differences in instantaneous water loss (EWL*i*) between species and through time, with a weak interaction between both factors (Tables 2 and 3). Post-hoc Duncan tests (*p* < 0.05) grouped *P. algirus* and *T. lepidus* having low rates and both *Podarcis* species having high rates, while *L. schreberi* occupied an intermediate position. EWL*i* also varied along time in all species, with both *Podarcis* species displaying higher temporal fluctuations (Fig. 2). When we added SVL and BM as covariates (ANCOVA-rm), interspecific differences were smoother but still significant, while temporal differences disappeared (Table 3).

**Figure 3:**
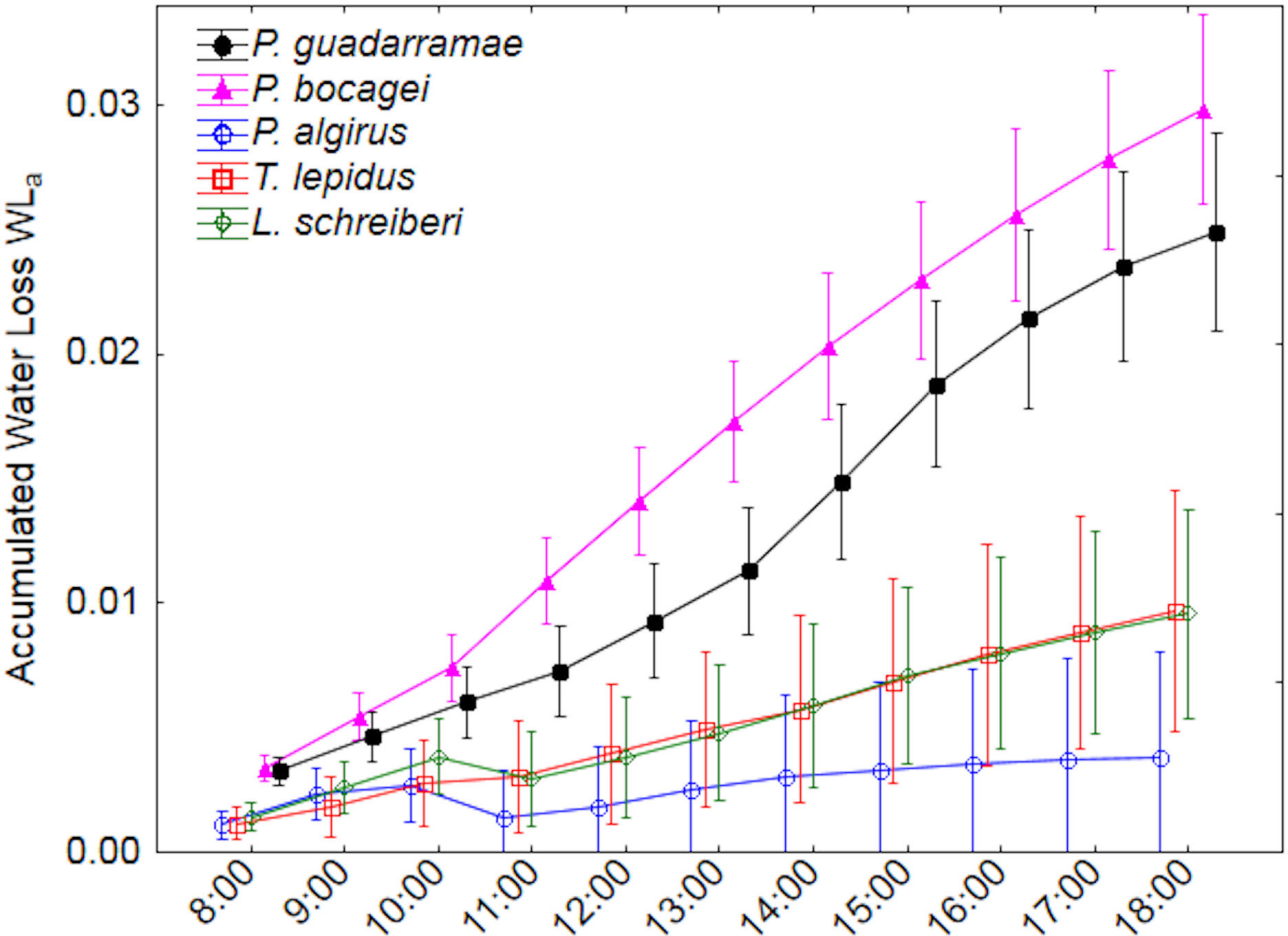
Accumulated water loss (EWL_*a*_) along a 12-hour experiment for five lizard species. Median values and 0.95 confidence intervals are displayed.

Accumulated water loss by evaporation (EWLa) revealed even more marked differences between the five species (Fig. 3). Here, two clear groups with no overlap could be distinguished; on one side the larger *T. lepidus* and *L. schreiberi* plus the medium-sized *P. algirus* all with low water loss rates and on the other side the small *P. guadarramae* and *P. bocagei* with much higher water loss rates (Duncan post-hoc tests *p* < 0.05). Again, after accounting for SVL and body mass, differences between species remain but differences in time intervals did not while the interaction between both factors was also conserved (Table 3). Neither in EWL*i* nor EWLa, did we observe interactions between factors and covariables.

###  Preferred temperatures vs. water loss rates

Mean *T*_*p*_ and EWL_*t*_ were inversely correlated between species while SVL and BM had no influence on the results (*n* = 5, *r*_partial_*T*_*p*_ −EWL_*t*_ = − 0.99, *T* = − 14.72, *P* = 0.04; *r*_partial_ SVL−EWL_*t*_ = − 0.94, *T* = − 2.70, *P* = 0.23; *r*_partial_ BM−EWL_*t*_ = 0.81, *T* = 1.41, *P* = 0.39). In contrast, within species EWL_*t*_ was positively correlated with SVL, negatively correlated with BM but independent from *T*_*p*_ for *L. schreiberi* (*n* = 5, *r*_partial_ *Tp*−EWLt = –0.62, *T* = − 1.59, *P* = 0.19; *r*_partial_ SVL−EWL_*t*_ = 0.84, *T* = 3.17, *P* = 0.03; *r*_partial_ BM−EWL_*t*_ = − 0.83, *T* = − 2.98, *P* = 0.04) and *P. algirus* (*n* = 5, *r*_partial_ *Tp* −EWLt = –0.39, *T* = 0.84, *P* = 0.45; *r*_partial_ SVL−EWL_*t*_ = 0.90, *T* = 4.02, *P* = 0.02; *r*_partial_ BM−EWL_*t*_ = − 0.86, *T* = − 3.38, *P* = 0.03). We did not detect significant relationships for the other three species.

## Discussion

The environmental differences between burnt and unburnt landscapes highlight the role of vegetation in the buffering of the natural fluctuations. Specifically, burnt microhabitats often used by lizards undergo larger daily variations of temperature and humidity, and also attain higher temperatures, especially in summer (Ferreira, 2015). When comparing lizards within the same trophic guild, this environmental contrast was expected to benefit Mediterranean lizards (compared to Atlantic lizards) from the thermal quality of open habitats created by fire regimes. We only have indirect support for this coming from two independent sources of evidence: (1) in southern France, fire recurrence increased the Mediterraneity (sensu Prodon, 1993) of the reptile community (Santos & Cheylan, 2013); and (2) in multiple localities, reptile species tend to be more common in burnt sites compared to unburnt ones (Santos & Poquet, 2010; Santos, Badiane & Matos, 2016). Although we hypothesised that these opposing responses would be caused by divergent ecophysiological features, our results only partially met our expectations that species favoured by fire should be more thermophile and economic in water loss. Certainly, the Mediterranean medium-sized *P. algirus* clearly selected for higher temperatures than the remaining species and lost less water than expected for its body size. However, the differences among the other species seem better explained by alternative factors such as refuge use, life history and body size/shape rather than by their responses to fire.

Thermal and hydric ecophysiology showed signs of a size/shape-independent trade-off across species but this should be confirmed by a formal analysis under the comparative method framework with an extended species dataset (Bauwens et al., 1995; Carneiro et al., in press). Within species, what we found was the influence of the surface/volume relationship (Schmidt-Nielsen, 1984) causing slender and smaller lizards to lose more water by body mass unit. Thus, in ecophysiological terms, species were not arranged in a Mediterranean-Atlantic axis. While the demographic responses to fire of these five lizards are mostly related to their biogeographic affinities and global distribution ranges (Sillero et al., 2009; Santos & Poquet, 2010; Santos & Cheylan, 2013; Ferreira, Mateus & Santos, 2016), the lack of complete concordance with their physiological features suggest a more complex scenario. This supports previous claims that the functional approach to predict responses of reptiles to fire is conceptually accurate but predictively weak (Smith et al., 2012; Smith, Bull & Driscoll, 2013).

*Psammodromus algirus* is considered a species with a wide ecological valence found from the border of Sahara in North Africa to mountain oak forests and other humid environments in the border of the Atlantic region in Iberia (Loureiro et al., 2008). Notwithstanding that previous studies reported high preferred temperatures (Bauwens et al., 1995), there is also evidence of activity under suboptimal thermal conditions (Carretero & Llorente, 1995). It is also the only species of the five studied with the body covered by keeled, overlapping scales (Arnold, 2002). Although this scale arrangement is likely the result from an adaptation for locomotion in bushy vegetation (matrix climbing, Arnold, 1987), it apparently provides protection against water loss acting as an exaptation (Gould & Vrba, 1982) when environmental humidity decreases. This may have given the species better conditions to survive in burnt areas. Although the short-term (one year after the fire) response of *P. algirus* can be negative in some Mediterranean landscapes, recovery has been reported after two years since fire has been reported (Santos, Badiane & Matos, 2016). Post-fire egg mortality (Smith et al., 2012) and life history of the species (Carretero & Llorente, 1997) may be underlaying reasons for these observed patterns.

Both large lizard species undoubtedly take advantage from lower water loss rates due to their lower surface/volume relationship (Schmidt-Nielsen, 1984). However, despite their opposite biogeographic affinities, they only differ slightly in hydric physiology which makes it difficult to interpret their responses to fire (Santos & Cheylan, 2013; Ferreira, Mateus & Santos, 2016). Instead, the variable responses of *T. lepidus* according to the population studied, and the decrease of *L. schreiberi* in response to fire intensification should be better interpreted in terms of habitat use. *Timon lepidus* is more mobile and tends to occupy areas dominated by rocky substrates using big rock holes as refuge (Castilla & Bauwens, 1992). In some localities, the species occupies long-unburnt (and structured) habitats (Santos, Badiane & Matos, 2016) whereas in others only appears in repeated-burnt ones (Santos & Cheylan, 2013). This may be due to shifts in other ecological resources (i.e., habitat, prey) but also to different fire ages (Nimmo et al., 2014). In contrast, *L. schreberi* has small home ranges and uses ecotonal bushy vegetation to thermoregulate, forage and hide (Salvador, 1988) keeping its distribution mostly outside of the range of fire. We suggest here that the divergent responses of both species to fire are likely habitat-mediated while shared thermal (and partly hydric) ecophysiology would result from evolutionary convergence in two long-term separated lacertid lineages (Arnold, Arribas & Carranza, 2007). Interestingly, competitive exclusion between green (*Lacerta* sp.) and ocellated lizards (*Timon* sp.) at a geographic level is suggested to have shaped the historical biogeography of both groups (Ahmadzadeh et al., 2016).

Particular habitat requirements may explain why the two species of wall lizards (*Podarcis* sp.) show opposing responses to fire but similar physiological features. The geographic ranges of *P. bocagei* and *P. gaudarramae* widely overlap geographically and both species are frequently found in syntopy (Carretero, 2008). However, field-work experience demonstrated that *P. guadarramae* is the only species found in repeatedly burnt spots (Ferreira, Mateus & Santos, 2016). This lizard is more associated to bare rocky substrates than *P. bocagei*, which uses a wider variety of substrates (Kaliontzopoulou, Carretero & Llorente, 2010), trend that is accentuated in syntopy (Gomes, Carretero & Kaliontzopoulou, 2016). Since mean preferred temperatures and water loss rates did not differ, we interpret the dominance of *P. guadarramae* after fire intensification as another result of different habitat and refuge use. In fact, head fattening of *P. guadarramae* might confer and advantage when rock crevices are used as main refuge (Kaliontzopoulou et al., 2012) as expected in burnt areas. Nevertheless, the accentuated diel variation in preferred temperatures by *P. bocagei* also suggest sensitivity to midday conditions either thermal or hydric, which might provide some support for a ecophysiological constrain when compared to *P. guadarramae*. This aspect should be explored in the future with continuous monitoring of individual lizards (Bowker, Wright & Bowker, 2010; Bowker, Bowker & Wright, 2013).

Fire is a fundamental driver of ecosystem functioning and composition in the Mediterranean basin (Blondel et al., 2010). Species that are mostly distributed in this bioregion occupy fire-prone landscapes with biota likely resulting from a long evolutionary association with fire (Pausas & Keeley, 2009). The effects of fire are observable at multiple scales from the landscape (variation in land cover) to the microhabitat (variation in temperature and humidity ranges). As ectotherms, the biological and ecological processes of reptiles are dependent on environmental temperature. However, heliothermic lizards are able to thermoregulate accurately if habitat complexity provides sufficient thermal heterogeneity for shuttling and selecting appropriate temperatures (Sears & Angilletta, 2015). Despite the sensitivity of reptiles to modifications in habitat structure (caused by fire), factors such as life history, microhabitat preferences and or thermoregulatory behaviour may have deviated results from a pure ecophysiological model. Further studies should be addressed to understand interactions between fire and other processes in order to more accurately predict reptile responses to fire-regimes using an extended species dataset. Meanwhile, current evidence suggests that ecophysiology plays a functional role in reptile responses to fire, which is likely habitat-mediated (Lindenmayer et al., 2008; Santos & Cheylan, 2013; Nimmo et al., 2014).

Although this is a first approach to a complex topic, ecophysiology already appears relevant in anticipating reptile responses to fire, even if this needs to be complemented by the analysis of other biological traits. Future studies should include more species and more regions to the analysis, not only to obtain better statistical support but also to allow phylogenetic correction in order to exclude the effects of long-term evolutionary history. Overall, our results already indicate that ecophysiology may provide mechanistic understanding for how species occurrence and abundance are spatially distributed at different geographic scales, and how they may be modified by human impacts (Sinervo et al., 2010; Huey et al., 2012; Lara-Reséndiz et al., 2015; Valenzuela-Ceballos et al., 2015).

##  Supplemental Information

10.7717/peerj.2107/supp-1 Table S1 Temperatures and body weights of lizards during lab experiments Preferred temepratures measured and body mass measurements of individuals of the five lizard species used during lab experiments. The first excel folder include the raw data and the second one varialbe explanation.

## Additional Information and Declarations

### Data Availability

The following information was supplied regarding data availability: The raw data has been supplied as a Supplemental Dataset.
